# Visual and Thermal Image Processing for Facial Specific Landmark Detection to Infer Emotions in a Child-Robot Interaction

**DOI:** 10.3390/s19132844

**Published:** 2019-06-26

**Authors:** Christiane Goulart, Carlos Valadão, Denis Delisle-Rodriguez, Douglas Funayama, Alvaro Favarato, Guilherme Baldo, Vinícius Binotte, Eliete Caldeira, Teodiano Bastos-Filho

**Affiliations:** 1 Northeast Network of Biotechnology (RENORBIO), Postgraduate Program in Biotechnology, Health Sciences Center, Federal University of Espirito Santo (UFES), Av. Marechal Campos, 1468, Vitoria-ES 29043-900, Brazil; 2 Postgraduate Program in Electrical Engineering, UFES, Av. Fernando Ferrari, 514, Vitoria-ES 29075-910, Brazil; 3 Center of Medical Biophysics, University of Oriente, Patricio Lumumba s/n, Santiago de Cuba 90500, Cuba; 4 Computer Engineering Department, UFES, Av. Fernando Ferrari, 514, Vitoria-ES 29075-910, Brazil; 5 Mechanical Engineering Department, UFES, Av. Fernando Ferrari, 514, Vitoria-ES 29075-910, Brazil; 6 Electrical Engineering Department, UFES, Av. Fernando Ferrari, 514, Vitoria-ES 29075-910, Brazil

**Keywords:** Viola-Jones, facial emotion recognition, facial expression recognition, facial detection, facial landmarks, infrared thermal imaging, homography matrix, socially assistive robot

## Abstract

Child-Robot Interaction (CRI) has become increasingly addressed in research and applications. This work proposes a system for emotion recognition in children, recording facial images by both visual (RGB—red, green and blue) and Infrared Thermal Imaging (IRTI) cameras. For this purpose, the Viola-Jones algorithm is used on color images to detect facial regions of interest (ROIs), which are transferred to the thermal camera plane by multiplying a homography matrix obtained through the calibration process of the camera system. As a novelty, we propose to compute the error probability for each ROI located over thermal images, using a reference frame manually marked by a trained expert, in order to choose that ROI better placed according to the expert criteria. Then, this selected ROI is used to relocate the other ROIs, increasing the concordance with respect to the reference manual annotations. Afterwards, other methods for feature extraction, dimensionality reduction through Principal Component Analysis (PCA) and pattern classification by Linear Discriminant Analysis (LDA) are applied to infer emotions. The results show that our approach for ROI locations may track facial landmarks with significant low errors with respect to the traditional Viola-Jones algorithm. These ROIs have shown to be relevant for recognition of five emotions, specifically disgust, fear, happiness, sadness, and surprise, with our recognition system based on PCA and LDA achieving mean accuracy (ACC) and Kappa values of 85.75% and 81.84%, respectively. As a second stage, the proposed recognition system was trained with a dataset of thermal images, collected on 28 typically developing children, in order to infer one of five basic emotions (disgust, fear, happiness, sadness, and surprise) during a child-robot interaction. The results show that our system can be integrated to a social robot to infer child emotions during a child-robot interaction.

## 1. Introduction

Child-Robot Interaction (CRI) is a subfield of Human-Robot Interaction (HRI) [1], which is defined as the interaction between humans and robotic systems. Inside the several possibilities of HRI and CRI, socially assistive robots are being used as a therapy-aid tool for children with Autism [2,3]. One feature that could improve this interaction is the ability of recognizing emotions, which can be used to provide a better CRI. For instance, children with autism spectrum disorder (ASD) tend to lack the ability of emotion display, thus, the robots should rely on involuntary biological signals measurements, such as skin thermography [4,5,6].

The face is a region of the body that has a high response to emotions, and the facial thermal print changes may be linked to the child emotion. Thus, this feature can be a useful parameter to be applied in a CRI, since this biological signal is not voluntary and not easily mutable [7]. Due to this feature, recent studies are focused on facial detection and thermography to evaluate emotion expressions in affective computing [8,9,10]. Moreover, it is a more comfortable and unobtrusive technique to evaluate emotions, since no sensor touching the child is needed, such as electrodes used in electroencephalography and electrocardiography [8,11,12].

A conventional system for facial emotion recognition is composed of the following three main stages: face and facial component detection, computation of various spatial and temporal features, and emotion classification [10]. Then, the first stage for face detection over an input image, and consequently to locate facial components (such as eyes, nose, and mouth) or landmarks of interest, is a crucial task and still a challenge. In fact, to accurately discriminate emotions, it is necessary to apply geometric or appearance features based methods [10,13,14,15], being the latter the most popular, due to its superior performance [16]. On the other hand, Facial Landmarks (FL) should be used to locate salient points of facial regions, such as the end of the nose, ends of the eye brows, and the mouth [10,16].

Many studies have demonstrated that dividing the face into specific regions for facial feature extraction can improve the performance during emotion recognition [17,18,19,20,21,22,23,24,25,26,27,28,29]. However, this strategy may be affected by improper face alignment. Moreover, other works based on learning [30,31] for feature extraction from specific face regions have been proposed to locate those facial regions with higher contribution for emotion recognition. Nevertheless, these approaches are difficult to be extended as a generic system, due to the fact that positions and sizes of the facial patches vary according to the training data.

It is worth commenting that studies using thermal cameras for emotion recognition have shown promising results, but low-cost thermal cameras typically present a poor resolution, making it difficult to accurately detect facial regions by applying conventional methods as the Viola-Jones algorithm, widely used on visual images [15,32].

Then, we hypothesized that a low-cost system for simultaneous capture of both visual and thermal cameras may increase the accuracy for locations of specific facial regions of interest (ROIs) over faces, and consequently improve the feature extraction, increasing the emotion discrimination. This way, we consider an alternative few-explored, which is to firstly apply Viola-Jones algorithm on the visual image to locate desired ROIs, and after transferring it for its corresponding thermal image, but including as a last stage a method for ROI location correction based on error probability, taking into account manual annotations of a trained expert over a reference frame.

Thus, the goal of this work is to propose a system able to detect facial ROIs for five emotions (disgust, fear, happiness, sadness, and surprise) in typically developing children during an interaction with a social robot (as an affective stimulus). In this study, our low-cost camera system allows obtaining pairs of synchronized images for detecting ROIs in the visual image using the Viola-Jones algorithm as a first stage, and then, transferring these ROIs to the corresponding thermal camera frame through a homography matrix. As the main novelty, we introduced here a new way to accurately improve the ROI locations after applying both Viola-Jones and homography transform. This approach computes the error probability to automatically find that ROI located over thermal images, which is better placed according to manual annotations of a trained expert. This ROI of highest probability (with lowest location error) is latter used to relocate other ROIs, improving the overall accuracy. Then, better appearance features can be extracted, in order to increase the emotion discrimination by our proposed recognition system. Similarly, this method may be extended to other studies aiming to accurately locate ROIs over facial thermal images, which are physiologically relevant, such as described in [17,21], allowing to understand phenomena linked to behaviours, emotions, stress, human interactions, among others. As a relevance of this work, our system is capable of detecting ROIs on the child’s face, which has neurophysiological importance for emotion recognition through thermal images recorded in an unobtrusive way. Additionally, methods for feature extraction and dimensionality reduction are applied on specific ROIs for emotion recognition using Linear Discriminant Analysis (LDA). As another highlight, a set of visual and thermal images is acquired in an atypical context in which a social robot is used as an emotional stimulus in an interaction with children, in order to test the proposed system for specific ROIs detection and emotion recognition. For our knowledge, this type of approach has not been explored in other studies.

This work is structured as follows. Section 2 presents a description of several works of the state-of-the-art. Section 3 presents a system for image acquisition, in addition to a proposal based on the Viola-Jones algorithm and error probability for facial ROI location. Moreover, the experimental protocol and methods for feature extraction, dimensionality reduction, and classification are described. Section 4 presents the experimental findings about the automatic method for ROI placement and children’s emotion recognition during the interaction with the robot. Afterwards, Section 5 presents the findings of this work and compare them to previous studies, summarizing also its main contributions and limitations. Finally, Section 6 presents the Conclusion and Future Works.

## 2. Related Works

Many research to recognize facial emotion by contact-free strategies have proposed automatic methods for both face and facial ROI detection over visual and thermal images, as constructing an effective face representation from images is a crucial step for successful automatic facial action analysis, in order to recognize facial emotions. In this field, there is the Facial Action Coding System (FACS), which is a taxonomy of human facial expressions designed to facilitate human annotation of facial behaviour [9,14,33]. For instance, a total of 32 atomic facial muscle actions, termed Action Units (AUs), and 14 additional descriptors related to miscellaneous actions are specified, which are widely used by automatic methods to locate facial landmarks and ROIs. These regions are used by methods based on geometric [9,10,15] and appearance features to discriminate emotions [9,14]. Appearance representations use textural information by considering the intensity value of the pixels, whereas geometric representations ignore texture and describe shape explicitly [9,14,15]. Here, we focused our revision of the state-of-the-art on approaches using only appearance features on the target face, which are generally computed by dividing the face region into regular grid (holistic representation). Appearance features can be obtained to encode low or high-level information. For example, low-level information can be encoded through low-level histograms that are computationally simple and ideal for real-time applications, Gabor representations, data-driven representations by applying bag-of-words, among others. Furthermore, higher level of information can be encoded through Non-Negative Matrix Factorization (NMF) [9]. However, the effectiveness of the feature extraction to increase the emotion discrimination may be affected by several factors, such as head-pose variations, illumination variations, face registration, occlusions, among others [9].

In [16] the authors used the Haar classifier for face detection, which is widely applied, due to its high detection accuracy and real time performance [32]. They extracted appearance features from the global face region by applying Local Binary Pattern (LBP) histogram that take care of minor changes of facial expression for different emotions [9,34], followed by Principal Component Analysis (PCA) for dimensionality reduction, to improve the speed of computation in real time during six emotions (anger, disgust, fear, happiness, sadness, and surprise). This approach is customizable person to person, and achieved an accuracy (ACC) of 97%. It is worth mentioning that unlike a global-feature-based approach, different face regions have different levels of importance for emotion recognition [17]. For example, the eyes and mouth contain more information than the forehead and cheek. Notice that LBP has been widely used in many research of emotion recognition. Refer to Ref. [34] for a comprehensive study about methods based on LBP for emotion recognition.

Another study [14] used specific regions for appearance feature extraction by dividing the entire face region into domain-specific local regions, using the landmark detection method presented in Ref. [35] that uses ensemble of regression trees. These authors used facial point locations to define a set of 29 face regions covering the whole face, which was based on expert knowledge regarding face geometry and AU-specific facial muscle contractions, such as shown in Ref. [33]. Ensemble of regression trees are used to estimate the face landmark locations directly from a sparse subset of pixel intensities, achieving super-real-time performance with high quality predictions. Similarly, they used LPB descriptor for appearance feature extraction, achieving an ACC of 93.60% after applying Support Vector Machine (SVM) with Radial Basic Function (RBF) kernel.

In Ref. [36], a comparative study of methods for feature extraction, such as Kernel Discriminant Isometric Mapping (KDIsomap), PCA, Linear Discriminant Analysis (LDA), Kernel Principal Component Analysis (KPCA), Kernel Linear Discriminant Analysis (KLDA), and Kernel Isometric Mapping (KIsomap) was conducted, achieving the best performance (ACC of 81.59% on the JAFFE database, and 94.88% on the Cohn-Kanade database) for KDIsomap during seven emotions (anger, joy, sadness, neutral, surprise, disgust and fear), but without significant difference compared with other approaches. Here, the authors used the well-known Viola-Jones algorithm to detect the face [32], which is suitable for real-time applications. This method uses a cascade of classifiers by employing Haar-wavelet features, which usually use the eye position detected in the face region to align the other detected face regions.

In Ref. [37] the authors propose the Central Symmetric Local Gradient Coding (CS-LGC) algorithm to define the neighborhood as a 5 × 5 grid, using the concept of center symmetry to extract the gradient information in four directions (horizontal, vertical, and two diagonals) for feature extraction over target pixels more representative. Afterwards, they also applied PCA for dimensionality reduction, followed by the Extreme Learning Machine (ELM) algorithm. The evaluation of this approach was conducted through JAFFE and Cohn-Kanade databases, which contain grayscale visual images related to the following emotions: anger, disgust, fear, happiness, neutral, sadness and surprise. Accuracies of 98.33% and 95.24% for Cohn-Kanade and JAFFE were obtained, respectively, being relatively better compared with other operators for feature extraction, such as LBP.

Several studies for emotion recognition have been conducted with two kinds of camera (one visual and another infrared), such as in Ref. [20]. Those authors proposed a fusion scheme by applying PCA over both thermal and visual faces for feature extraction, and k-nearest neighbors to recognize two classes (surprised and laughing) with mean ACC of 75%. Additionally, in Ref. [23] a comparison for emotion recognition using visual and infrared cameras was carried out and four typical methods, including PCA, PCA plus LDA, Active Appearance Model (AAM), and AAM-based plus LDA were used on visual images for feature extraction, whereas PCA and PCA plus LDA were applied on infrared thermal images using four ROIs (forehead, nose, mouth, and cheeks). These authors used k-nearest neighbors to recognize six emotions (sadness, anger, surprise, fear, happiness, and disgust). It is worth mentioning that the eye locations over the thermal were manually performed for those authors, which latter were used to locate the aforementioned four ROIs. In Ref. [22] an interesting approach using both kinds of camera was addressed, including the use of eyeglasses, which are opaque to the thermal camera, but visible to the visual camera.

Another interesting work shows that infrared and visual cameras can be combined into a multi-modal sensor system to recognize fear [24], through electroencephalogram (EEG) signals, eye blinking rate, and facial temperature while the user watched a horror movie. An Adaptive Boosting (AdaBoost) algorithm was used to detect the face region, and a geometric transform to make the coordinates of the two images (visible-light and thermal) coincident was used. Similarly, other study was conducted on Post traumatic Stress disorder (PTSD) patients to infer fear through visual and thermal images [38]. In Ref. [39], an algorithm for automatic determination of the head center in thermograms was proposed, which has demonstrated to be sensitive to the head rotation or position. In Ref. [40], the authors proposed an unsupervised Local and Global feature extraction for facial emotion recognition through thermal images. For this purpose, they used a bimodal threshold to locate the face for feature extraction by PCA, after applying a method based on clustering to detect points of interest; for facial expression classification, a Support Vector Machine Committee was used. In Ref. [17], the face was extracted on thermal images after applying both median and Gaussian filters with further binarization to convert the gray scale image into pure black and white, and removing small sets of non-connected pixels to enhance the image quality. Afterwards, appearance features were extracted on defined ROIs over the thermal images, followed by Fast Neighbourhood Component Analysis (FNCA) and LDA for feature selection and recognition of five emotions, respectively.

More details about different methods for feature extraction, dimensionality reduction, feature selection, and classification can be reviewed in some studies [37] and also in extensive reviews, such as in Refs. [9,10].

The next section presents our proposed system for five emotions recognition, which allows accurately locating facial ROIs over thermal images, improving the appearance feature extraction.

## 3. Materials and Methods

### 3.1. Experimental Procedure

Seventeen typically developing children, 9 boys and 8 girls (aged between 8 and 12 years) participated in this study, who were recruited from elementary schools in Vitoria-Brazil. All had their parents’ permission, through signatures of Terms of Free and Informed Consent. In addition, children signed a Term of Assent, informing their wish in participating. This study was approved by the Ethics Committee of Federal University of Espirito Santo (UFES)/Brazil, under number 1,121,638. The experiments were conducted in a room within the children’s school environment, where the room temperature was kept between 20 ${}^{\circ }$C and 24 ${}^{\circ }$C, using a constant luminous intensity, such as done by Ref. [39].

**Figure 1:**
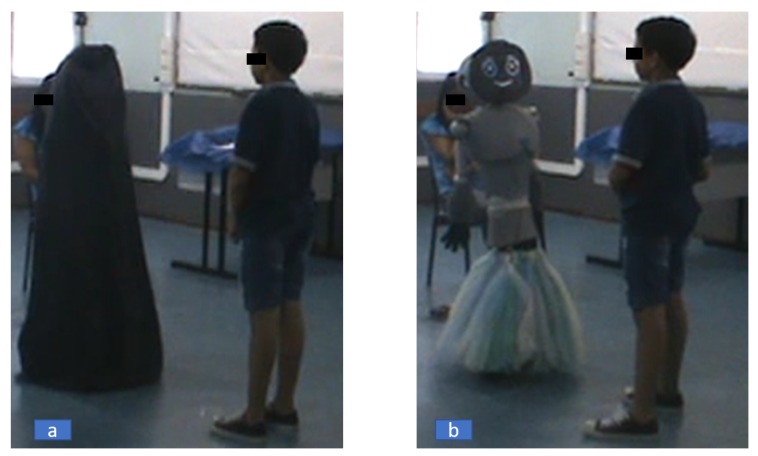
Experimental setup showing the child-robot interaction. (**a**) Before showing the robot; (**b**) After presenting it.

A mobile social robot (see Figure 1b), called N-MARIA (New-Mobile Autonomous Robot for Interaction with Autistics), built at UFES/Brazil to assist children during social relationship rehabilitation, was used in our research. This robot has attached a camera system to record facial images during interaction with children. More details about N-MARIA are given in Section 3.2.1.

The experiment was conducted in three phases, as follows. First, N-MARIA was initially covered at the room with a black sheet, except its attached camera system, that was turned on to record visual and thermal images of the frontal view with sampling rate at 2 fps, for further processing. Afterwards, the child was invited to enter the room, and sit comfortably for explanations about the general activities related to the experiment, being conditioned to a relaxed state for a period of time minimum of 10 min, in order to adapt her/his body to the temperature of the room, allowing her/his skin temperature to stabilize for baseline recordings, according to similar studies carried out in Refs. [21,41]. Once they had completed the relaxation period, the child was placed in front of the covered robot about 70 cm away from it, remaining in standing position. Immediately, recordings of the child face by the camera system were carried out for a period of one minute with the robot covered, one minute with the robot uncovered, and three minutes of interaction with the robot. After, the child spent two minutes answering a questionnaire about the experiment.

The first part of the recording (robot covered) corresponds to the experimental stage, called Baseline, whereas the next stage, presenting the uncovered robot is called Test. Before the robot is uncovered, the child was asked to permanently look forward without sudden facial movements or touching the face, avoiding any facial obstruction during video recordings.

After the removal of the black sheet that covered the robot, the first dialogue (self-presentation) of the robot was started. In addition to the self-introduction to the child, prompt dialogues during the experiment were related to questions, positive reinforcement and invitations. In the interaction with the child, that lasted two minutes, the child was encouraged to make communication and tactile interaction with the robot. At the end of the experiment, the child was again invited to sit and answer a structured interview about her/his feelings before and after seeing the robot, and also about the robot structure (if the child liked it, what she/he liked more, and what the child would change about it).

### 3.2. Contact-Free Emotion Recognition

**Figure 2:**
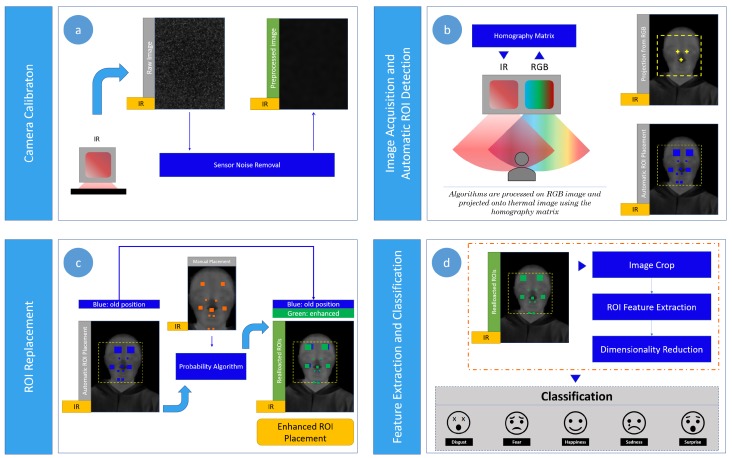
Overview of the proposed system for emotion recognition during a child-robot interaction. (**a**) Camera calibration; (**b**) image acquisition and automatic region of interest (ROI) detection; (**c**) ROI replacement; (**d**) feature extraction followed by the dimensionality reduction and emotion classification.

Figure 2 shows the proposed contact-free system for emotion recognition, which is composed of the following four steps: (a) camera calibration; (b) image acquisition and automatic ROI detection; (c) ROI replacement; (d) feature extraction followed by the dimensionality reduction and emotion classification.

Figure 2a shows a first stage to calibrate the camera system by obtaining a homography matrix to map the pixels of the visual camera image into the thermal camera image, considering the relative fixed position between the two cameras. Also, another process is performed to obtain a frame that contains intrinsic noise of the infrared sensor, which is latter used in a second stage (Figure 2b) to remove the sensor noise (inherent to the camera) over the current thermal image captured. In this second stage, the image acquisition process is carried out taking synchronous images from both visual and infrared cameras, which are pre-processed to enhance the automatic facial ROIs detection by applying the Viola-Jones algorithm on the visual image. Then, the ROIs placed on the visual image are projected into the thermal image using the homography matrix. As a third stage, manual annotations by a trained expert over a reference frame are used to accurately relocate the ROIs by applying our approach based on errors of probability, such as shown in Figure 2c. Afterwards, feature vectors related to thermal variations are computed on the detected ROIs, and after reduced by applying PCA for dimensionality reduction for five emotion recognition in a last stage by LDA. More details about the proposed recognition system are given in the next subsections.

#### 3.2.1. Camera System and N-MARIA

**Figure 3:**
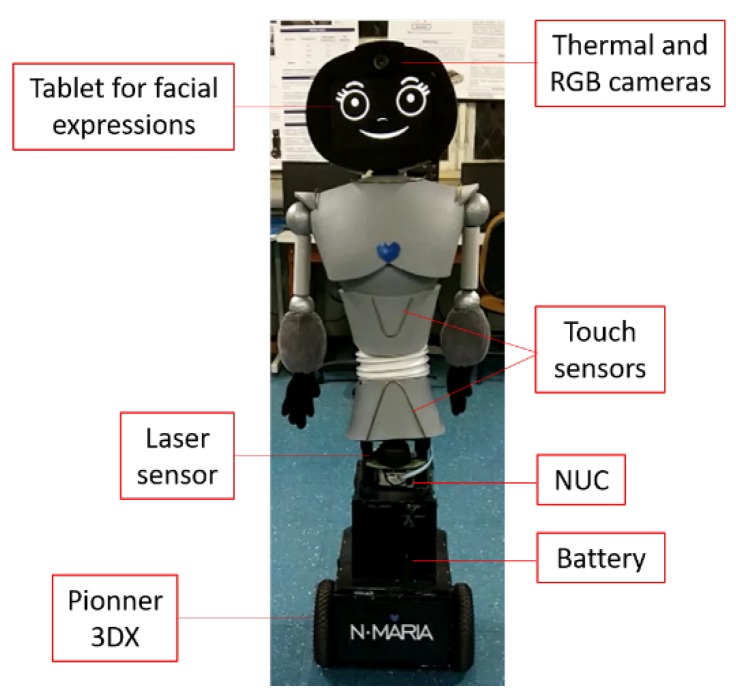
N-MARIA (New-Mobile Robot for Interaction with Autistics) developed at Federal University of Espirito Santo (UFES)/Brazil.

The camera system composed of both visual and thermal cameras was attached to the head of the social robot, such as shown in Figure 3. These two cameras were fixed so that both had approximately the same visual field. To capture thermal variations, a low-cost camera (Therm-App^®^) was used, which has spatial resolution of 384 × 288 ppi, frame rate at 8.7 Hz and temperature sensitivity <0.07 ${}^{\circ }$C. The normalization of the thermal images acquired in gray scale consisted of a brightness rate ranging from 0 to 255, where darker pixels correspond to lower temperatures, and lighter pixels correspond to higher temperatures. Moreover, a C270 HD Webcam (Logitech) was used to obtain visual images in RGB format, with a resolution of 1.2 MP.

The robot was built 1.41 m tall, considering the standard height of 9–10-year-old children. Additionally, soft malleable materials were used on the robot’s structure for protection of both children and internal robot devices. The Pioneer 3-DX mobile platform was responsible by locomotion, a 360${}^{\circ }$ laser sensor was used to locate the child in the environment, and a tablet was used as the robot face to display seven dynamic facial expressions during the robot-child interaction. Those expressions could also be remotely controlled through another tablet by the therapist, who could also control the robot behavior, expressions and dialogues emitted by the speakers.

#### 3.2.2. Camera Calibration

The camera calibration is done through a synchronous acquisition between visual and thermal images, and using a chessboard built with aluminum and electrical tape positioned in several possible angles. Then, the images obtained are processed with OpenCV calibration software [42], which uses Direct Linear Transform (DLT) to return a homography matrix [43], allowing transformation of points from the visual image to the thermal image in a robust way [32]. It is worth mentioning that there is not a homography matrix matching exactly points in all regions of the face (as they are not in the same plane), but the matrix obtained by DLT is used as an efficient approximation.

Also, other procedure to remove the intrinsic thermal noise of infrared sensors is carried out [44], which increases the quality of thermal images by correcting undesirable offsets. For that, a reference of an object with uniform body temperature covering the visual field of the thermal camera is recorded, which contains the intrinsic noise of the infrared sensor. Thus, it was expected to have a frame with the same brightness for all pixels, however, that did not occur. Thus, the frame with the intrinsic sensor noise was used in the pre-processing stage to eliminate the thermal noise, such as described in the next section.

#### 3.2.3. Image Acquisition and Pre-Processing

The thermal camera has maximum acquisition capacity of 8.7 fps whereas the visual camera has maximum capacity of 30 fps. Thus, to obtain temporal consistency, both visual and thermal images were simultaneously recorded with a sampling rate of 2 fps, which was suitable for our purposes.

During acquisition, the frame with the intrinsic sensor noise obtained in the Calibration stage was used to remove the intrinsic thermal noise of the current image acquired by subtracting pixel to pixel. Finally, a median filter was used to reduce salt and pepper noise from the thermal image.

#### 3.2.4. Face Landmark Detection

**Figure 4:**
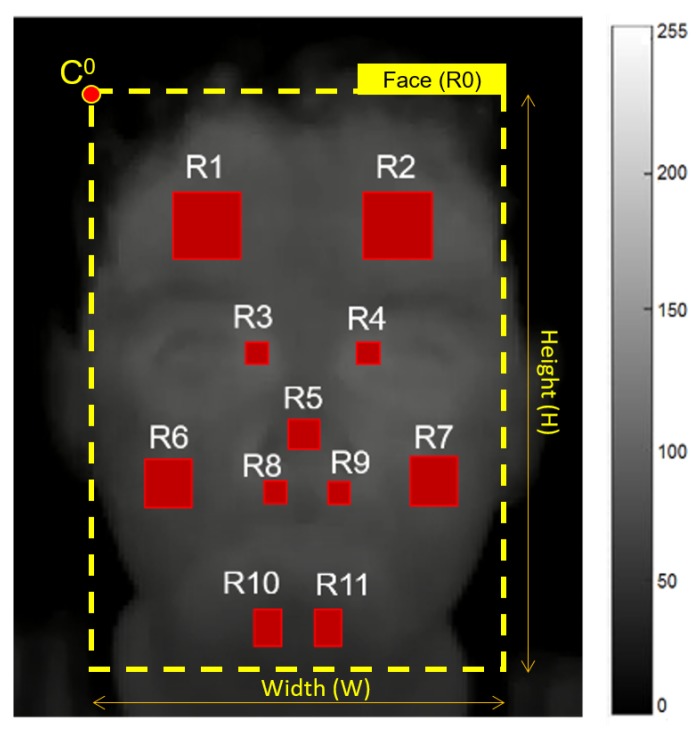
Facial ROIs. $R^{1}$, right forehead side; $R^{2}$, left forehead side; $R^{3}$, right periorbital side; $R^{4}$, left periorbital side; $R^{5}$, tip of nose; $R^{6}$, right cheek; $R^{7}$, left cheek; $R^{8}$, right perinasal side; $R^{9}$, left perinasal side; $R^{10}$, right chin side; $R^{11}$, left chin side.

An automatic method was proposed here for face landmark detection over a given set of frames $\left(I=\left\{i_{1},i_{2},\cdots ,i_{b},\cdots ,i_{B}\right\}\right)$, taking as reference annotated ROIs by a trained expert on the frame $i_{A}\left(b=A\right)$, such as shown in Figure 4. It is possible to observe that these manual annotations were located on eleven ROIs ($R_{A}=\left\{R_{A}^{1},R_{A}^{2},\cdots ,R_{A}^{k},\cdots ,R_{A}^{11}\right\}$) of thermal images, taking into account the relevance of these ROIs in other studies for facial emotion recognition [17,21]. Here, the facial ROI sizes were computed in the same way as in Refs. [17,18,21], using the width of the head ROI and the following defined proportions [18]: 6.49% for nose, 14.28% for forehead, 3.24% for periorbital region, 9.74% for cheek, 3.24% for perinasal region, and 5.19% for chin [17].

#### 3.2.5. Automatic ROI Detection

Infrared images are more blurred than color images [23], therefore the ROI detection over thermal images of low-cost cameras is a challenge. For this reason, the well-known Viola-Jones algorithm [32] was used on color images for head detection and other facial regions, such as nose and eyes [17,21,32]. Then, these initial detected regions were used as references to automatically locate eleven ROIs within the face (see Table 1), namely the nose, both sides of forehead, cheeks, chin, periorbital area (close to the eyes) and perinasal area (bottom of the nose).

In our study, the facial ROI sizes were also computed using the width of the head, and the aforementioned proportions [17,18,21]. Additionally, the facial ROIs were spatially placed, taking as reference the expert annotation. Afterwards, the corresponding facial ROIs were projected on the thermal image through the aforementioned transformation using a homography matrix (see Section 3.2.2), such as shown in Figure 4. Here, the ROI set of a thermal frame *b* is defined by $R_{b}=\left\{R_{b}^{0},R_{b}^{1},\cdots ,R_{b}^{k},\cdots ,R_{b}^{11}\right\}$, being **R**${}_{b}^{0}$ the head ROI, and **R**${}_{b}^{k}$ for k= 1 to 11 the facial ROIs. Notice that **R**${}_{b}^{k}$ is described by several pixels $R_{ij}$ for a range from 0 to 255 (gray scale of 8 bits).

Figure 2b,c show our proposal to accurately locate facial ROIs, formed by the following two-stages: (1) automatic ROI detection and (2) ROI placement correction.

#### 3.2.6. ROI Location Correction

A new method is proposed here to correct with accuracy the detected ROIs by the Viola-Jones algorithm, taking into account all pre-defined ROIs positions, which were manually annotated on a first frame by a trained expert.

Let **R**${}_{b}^{k}$ be an automatic detected ROI over the thermal frame $i_{b}$ that presents a coordinate $C_{b}^{k}=\left(C_{bx}^{k},C_{by}^{k}\right)$ at the left upper corner, which corresponds to **R**${}_{A}^{k}$ (annotated ROI over $i_{A}$) with coordinate $C_{A}^{k}=\left(C_{Ax}^{k},C_{Ay}^{k}\right)$ at the left upper corner too. Then, two probabilities $p_{bx}^{k}$ and $p_{by}^{k}$ can be calculated for **R**${}_{b}^{k}$, taking into account the expert annotation, such as described in Equations (1) and (2). These $p_{bx}^{k}$ and $p_{by}^{k}$ values are computed in relation to **x** and **y**, respectively. They take values closer to 1 if **R**${}_{b}^{k}$ location highly agrees with the manual annotation of the trained expert, which are shown in Equations (1) and (2). Notice that **R**${}_{b}^{k}$ is automatically obtained by applying the Viola-Jones algorithm, fixing defined proportions (see Section 3.2.4).

(1) $$p_{bx}^{k}=\frac{\exp(-|\frac{C_{bx}^{k}}{W_{b}}-\frac{C_{Ax}^{k}}{W_{A}}\left|\right)}{\sum_{i=1}^{11}\exp(-|\frac{C_{bx}^{i}}{W_{b}}-\frac{C_{Ax}^{i}}{W_{A}}\left|\right)},$$

(2) $$p_{by}^{k}=\frac{\exp(-|\frac{C_{by}^{k}}{H_{b}}-\frac{C_{Ay}^{k}}{H_{A}}\left|\right)}{\sum_{i=1}^{11}\exp(-|\frac{C_{by}^{i}}{H_{b}}-\frac{C_{Ay}^{i}}{H_{A}}\left|\right)},$$

(3) $$p_{b}^{k}=\min\left\{p_{bx}^{k},p_{by}^{k}\right\},$$

where *k* refers to the current facial ROI for analysis, taking values from 1 to 11; $W_{b}$ and $H_{b}$ are the width and height of **R**${}_{b}^{0}$ (head ROI for **i**${}_{b}$), respectively; $W_{A}$ and $H_{A}$ are the width and height of **R**${}_{A}^{0}$ (head ROI for **i**${}_{A}$); $p_{bx}^{k}$ and $p_{by}^{k}$ are the probabilities that **R**${}_{b}^{k}$ were correctly located on **i**${}_{b}$ regarding the trained expert annotation, in relation to *x* and *y* axes, respectively.

Finally, **R**${}_{b}^{k}$ (for which **C**${}_{b}^{k}$ is denoted as **C**${}_{b}^{ref}$) of lower probability $p_{b}^{k}$ is selected as a reference to correct the location for the other ROIs, using Equations (4) and (5). It is worth mentioning that **C**${}_{A}^{ref}$ notation is used for the annotated frame $i_{A}$.

(4) $$C_{bx}^{k^{\prime }}=\frac{C_{bx}^{ref}+\left(C_{Ax}^{k}-C_{Ax}^{ref}\right)}{W},$$

(5) $$C_{by}^{k^{\prime }}=\frac{C_{by}^{ref}+\left(C_{Ay}^{k}-C_{Ay}^{ref}\right)}{H},$$

where $C_{b}^{k^{\prime }}=\left(C_{bx}^{k^{\prime }},C_{by}^{k^{\prime }}\right)$ is the coordinate of the left upper corner for **R**${}_{b}^{k}$ relocated.

#### 3.2.7. Feature Extraction

Given a thermal frame $i_{b}$ formed by a set of ROIs, $R_{b}=\left\{R_{b}^{1},R_{b}^{2},\cdots ,R_{b}^{k},\cdots ,R_{b}^{K}\right\}$, being K=11 the total number of ROIs, it is possible to extract from $R_{b}$ a feature vector $F_{b}=\left\{f_{b}^{1},f_{b}^{2},\cdots ,f_{b}^{k},\cdots ,f_{b}^{K}\right\}$ that describes a pattern related to an emotion, being $f_{b}^{k}=\left\{f_{b1}^{k},f_{b2}^{k},...,f_{b14}^{k}\right\}$ features of **R**${}_{b}^{k}$. Table 2 presents the features adopted in our study, which agree with [17].

$R_{b}^{k}$ is the current ROI for feature extraction, $\bar{R_{b}^{k}}$ is the average value of **R**${}_{b}^{k}$, $\sigma_{b}^{2k}$ is the variance of **R**${}_{b}^{k}$, and $f_{b(c+7)}^{k}$ for *c* equal 1 to 7 are other seven features corresponding to the difference of computed features throughout consecutive frames. So we have eleven ROIs (see Figure 4), and 14 features for each of them. This gives a set of 154 features per frame.

#### 3.2.8. Dimensionality Reduction and Emotion Classification

Let $T=\left(F_{1},y_{1}\right),\left(F_{2},y_{2}\right),\ldots ,\left(F_{b},y_{b}\right),\ldots ,\left(F_{n},y_{n}\right)$ be the training set, where *n* is the number of samples, and $F_{i}$ is a *d*-dimensional feature vector with class label $y_{b}\in 1,2,\ldots ,5$. PCA method based on Single Value Decomposition [9,16,20,23,36] is applied on $F_{i}$ to obtain the principal component coefficients, which are used in both training and validation sets to reduce the set of 154 features, in order to allow a robust and fast emotion recognition. As advantages, PCA is few sensitive to different training sets, and it can outperform other methods as LDA when the training set is small [45]. This method has been successfully used in many studies to represent, in lower dimensional subspace, the high dimensional feature vectors, which are obtained by applying appearance-based methods [16,23]. It is worth mentioning that before applying PCA in our study, the feature vectors of the training set were normalized using both mean and standard deviation values as reference. Then, the validation was normalized using the same reference values (mean and standard deviation) obtained from the training set.

Some classifiers, such as LDA [17,23,46] and Quadratic Discriminant Analysis (QDA) [12,47] by applying full and diagonal covariance matrices, as well as other three classifiers, such as Mahalanobis discrimination [12,48], Naives Bayes [49], and Linear Support Vector Machine (LSVM) [12,14,50] are used in our study to assign objects to one of several emotion classes based on a feature set.

### 3.3. Statistical Evaluation

From images recorded during both moments of the experiment (Baseline and Test), a set of 220 thermography frames randomly selected from 11 children was annotated by a trained expert, selecting the ROIs defined in Figure 4. These annotated images were used as reference to evaluate, through Euclidean distances (see Equation (6)), the accuracy and precision of both Viola-Jones without ROI relocation, and Viola-Jones applying our ROI relocation algorithm.

(6) $$D=\sqrt{{\left(A_{x}-M_{x}\right)}^{2}+{\left(A_{y}-M_{y}\right)}^{2}},$$

where $\left(A_{x},A_{y}\right)$ is the coordinate obtained by the automatic method, and $\left(M_{x},M_{y}\right)$ is the coordinate obtained by the manual method. When *D* is close to zero, that means high accuracy.

The statistical analysis used for comparison between both approaches for each ROI was the Wilcoxon Signed Rank Test for zero median.

In order to evaluate our proposed system for emotion recognition, a published database (available in the supporting information of [17] at the website —Available at https://journals.plos.org/plosone/article?id=10.1371/journal.pone.0212928) was used, which is formed by feature vectors labeled as the following five emotions: disgust, fear, happiness, sadness and surprise. This database was collected on 28 typically developing children (age: 7–11 years) by an infrared thermal camera [17]. It is worth noting that this database was also created with children of similar age range, using the same thermal camera and feature set (a total of 154 features) described in our study (see Section 3.1, Section 3.2.2 and Section 3.2.7), and computing this feature set over the ROIs defined on Figure 4. Notice that the correct locations of these ROIs were visually inspected by a trained expert. For this reason, it was possible to compare the recognition system using one of the following methods: PCA for dimensionality reduction and Fast Neighbor Component Analysis (FNCA) [17] for feature selection. Here, the training and validation sets were chosen for several runs of cross-validation (kfold=3), and metrics such as accuracy (ACC), Kappa, true positive rate (TPR), and false positive rate (FPR) were used [51].

On the other hand, this published database was used to train our proposed system based on PCA, but only using data collected from those children that presented ACC higher than 85% during the emotion recognition [17]. Then, our trained system was used to infer the children emotion during our experimental protocol described in Section 3.1.

## 4. Results

### 4.1. Automatic ROI Location

**Figure 5:**
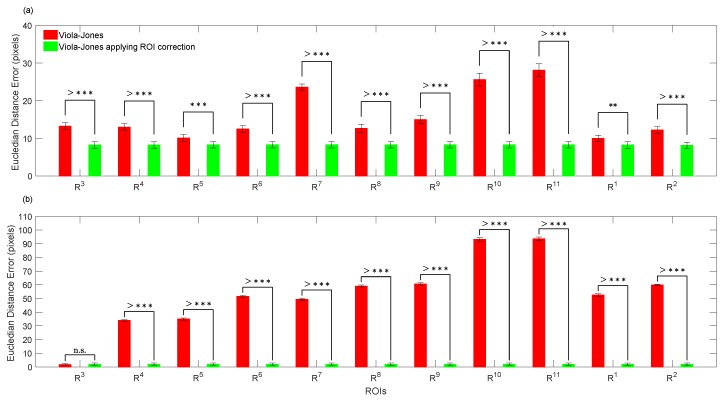
Comparison between Viola-Jones (without applying ROI relocations) and Viola-Jones applying ROI relocations, computing the mean and standard error per ROIs: (**a**) analysis from Baseline; (**b**) analysis from Test. n.s means no significant difference (p>0.05), while * (p<0.05), ** (p<0.01), *** (p<0.001) and >*** (p<0.0001) indicate significant difference. **R**${}^{1}$, right forehead side; **R**${}^{2}$, left forehead side; **R**${}^{3}$, right periorbital side; **R**${}^{4}$, left periorbital side; **R**${}^{5}$, tip of nose; **R**${}^{6}$, right cheek; **R**${}^{7}$, left cheek; **R**${}^{8}$, right perinasal side; **R**${}^{9}$, left perinasal side; **R**${}^{10}$, right chin side; **R**${}^{11}$, left chin side.

The performance of the proposed method using trained expert criteria and error probability to accurately detect face landmarks was validated on 11 children through two sets of 220 annotated thermal frames by the trained expert, each one obtained for the following two conditions: (1) Baseline, (2) Test (see Section 3.1). Figure 5a,b shows that our proposal significantly improved the ROI placements regarding trained expert criteria. For both conditions, the proposed method closely agreed (errors lower than 10 pixels) with the trained expert, although the children trended to make abrupt face movements for the second condition, as they may have got surprised by the uncovered robot. However, the Viola-Jones algorithm, non-assisted by the error probability, significantly disagreed with the trained expert, such as shown in Figure 5a,b. Notice that Viola-Jones presented the highest error locating both **R**${}^{10}$ and **R**${}^{11}$, which corresponds to the right and left chin sides, respectively. This undesirable mistakes may have been caused by mouth movements during talking or by facial expressions, such as happiness and surprise. As a highlight, our proposal takes into account the best located ROI, **R**${}^{1}$ or **R**${}^{5}$ for Baseline, and **R**${}^{3}$ for Test, which reduced the location error as much as possible, such as shown in Figure 5a,b, respectively. It is worth mentioning that our approach for ROI replacements uses the probability error to select (taking into account the manual annotations of a trained expert) that ROI better located during the first stage for automatic ROI locations by applying Viola-Jones. Then, this selected ROI is used to relocate the other neighbor ROIs. Therefore, the first stage using Viola-Jones to detect the three initial ROIs is quite decisive.

**Figure 6:**
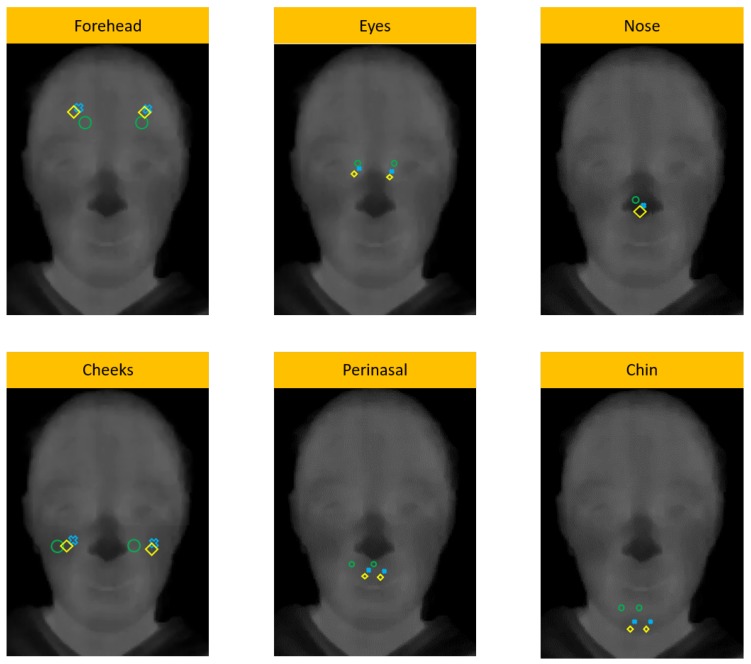
Comparison between manual placement (in yellow, used as reference), Viola-Jones algorithm (green) and Viola-Jones with our replacement algorithm (blue). Such as shown, our replacement algorithm obtained better results than using only Viola-Jones algorithm.

Figure 6 shows, for a child, the ROI placement using the automated Viola-Jones algorithm (green), the recalculated algorithm (blue) and the manual placement (yellow). In Ref. [17,21], the authors demonstrated that face regions (see Figure 4) linked to the set of branches and sub-branches of vessels that innervate the face are decisive to study emotions, as the skin temperature variations over these regions can be measured by IRTI. Figure 6 shows that our proposal based on probability error can be used to supervise automatic methods for ROI locations, in order to improve the appearance feature extraction over detected ROIs and, consequently, increase the performance during the emotion recognition.

### 4.2. Emotion Recognition

**Figure 7:**
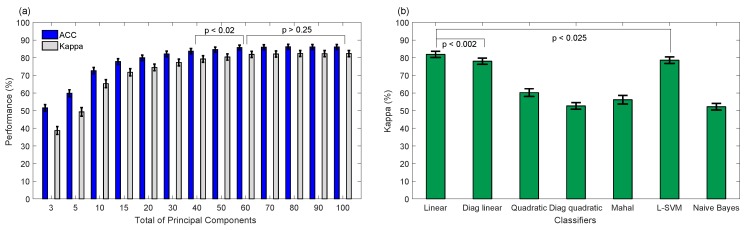
Performance of classification of five emotions applying principal component analysis (PCA) for dimensionality reduction. (**a**) Average accuracy and Kappa obtained by applying PCA for different principal components plus Linear Discriminant Analysis for classification; (**b**) average Kappa achieved for 60 principal components plus other classifiers.

A database with 28 typically developing children was used to analyze the performance of the proposed system for five emotions recognition [17]. This database contains appearance feature vectors that were computed on eleven face ROIs (see Figure 4), whose correct placements were carefully verified by a trained expert. Then, the effectiveness of PCA plus other classifiers for five emotions classification was evaluated here, being PCA a popular adaptive transform that under controlled head-pose and imaging conditions may be useful to capture features of expressions efficiently [9]. Figure 7a shows the average performance by applying PCA for different principal components plus LDA classifier based on full covariance matrix, being 60 components sufficient for five emotions recognition. Notice that non-significant difference (p<0.05) using more than 60 components was achieved. Similarly, non-significant difference was obtained using PCA with 60 components plus other classifiers, such as LDA using full (Linear) or diagonal (Diag Linear) covariance matrices, and Linear SVM, such as shown in Figure 7b. For instance, LDA using full and diagonal covariance matrices achieved Kappa values of 81.84 ± 1.72% and 77.99 ± 1.74%, respectively, and 78.58 ± 1.83% for Linear SVM. LDA based on full covariance matrix has low-computational cost and can be easily embedded into the N-MARIA hardware for on-line emotion recognition during the child-robot interaction. Moreover, PCA and LDA have been successfully used in other similar studies [16,17,20,23,36], achieving promising results. Then, we selected PCA with 60 principal components plus LDA based on full covariance matrix as the best setup for five emotions recognition.

**Figure 8:**
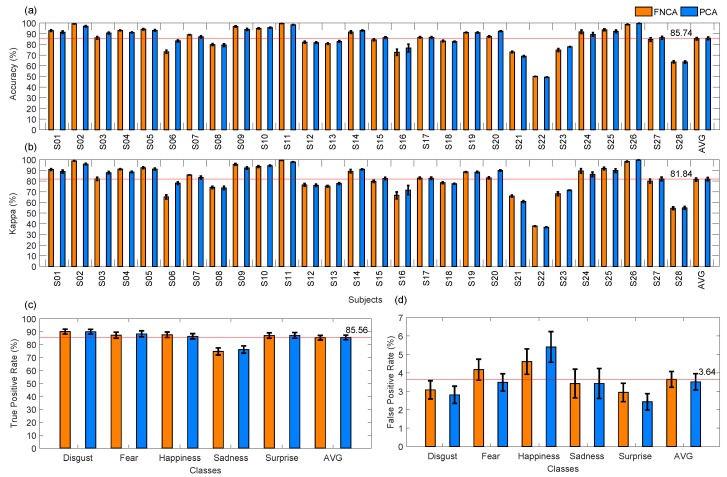
Performance of classification of five emotions applying principal component analysis (PCA) and Fast Neighbourhood Component Analysis (FNCA) for dimensionality reduction.

Consequently, a comparison between PCA using 60 components and FNCA was carried out to recognize five emotions of the 28 typically developing children, using LDA based on full covariance matrix. Figure 8 and Table 3 and Table 4 show that both PCA and FNCA methods are interchangeable for dimensionality reduction, achieving mean ACC of 85.29% and 85.75%, respectively.

It is worth noting that PCA improved the performance for subject S06 (ACC of 83.27% and Kappa of 78.10% for PCA, and ACC of 73.11% and Kappa of 65.09% for FNCA), such as shown in Figure 8a,b. This improvement may be consequence of the PCA robustness for feature extraction under controlled head-pose and imaging conditions [9]. Also, notice that for subject S22 the lowest performance was achieved by applying PCA (ACC of 49.27% and Kappa of 36.75%) and FNCA (ACC of 49.98% and Kappa of 37.78%). However, ACC values higher than 85.74% were achieved for a total of 14 children by applying PCA or FNCA, being the highest ACC of 99.78% for subject S26 using PCA. Labelling feature vectors of emotion patterns is a challenge, mainly when labels are assigned over periods of time, as some delays between both perception and manual label assignment may exist. Similarly, a visual stimulus may produce different facial emotions, thus, the manual labeled procedure is subjective. Then, an automatic method for feature set labelling may be suitable to obtain reliable labels and therefore a classification model for LDA.

On the other hand, Figure 8c shows that four emotions (disgust, fear, happiness, and surprise) were successfully recognized by applying both PCA and FNCA, achieving TPR > 85%, whereas sadness was classified with less sensitivity (TPR of 74.69% and 76.15% for FNCA and PCA, respectively) by the proposed system. As a highlight, low values of FPR (≤5.40%) were obtained to recognize all studied emotions. In general, the results achieved show that PCA and FNCA are interchangeable to obtain the features that increase the emotion discrimination by discovering those regions that are informative in terms of expressions [9]. Similar to other studies [52], the sadness emotion was less recognized (ACC < 77%) than the other emotions, as each child expressed sadness in a different manner, producing a large intra-class variability for sadness.

Many researchers [1] have conducted studies to infer facial emotions over visual images using only three out of the six basic expressions, specifically happiness, surprise and sadness, as these emotions are easier to be recognized for humans, and have been successfully used to generate cohesive expressions across participants. Similarly, other studies using visual images report that the recognition rates for expressions as fear or disgust can be very low, in a range from 40 to 50%. As a highlight, five basic emotions were recognized by our proposed system based on infrared camera, achieving the highest performance for both disgust (TPR of 89.93%) and fear (TPR of 88.22%), followed by happiness (TPR of 86.36%) and surprise (TPR of 87.14%), such as shown in Figure 8c and Table 4. In agree with other studies [17,21], our results show that thermal images are promising for facial emotion analysis and recognition.

**Figure 9:**
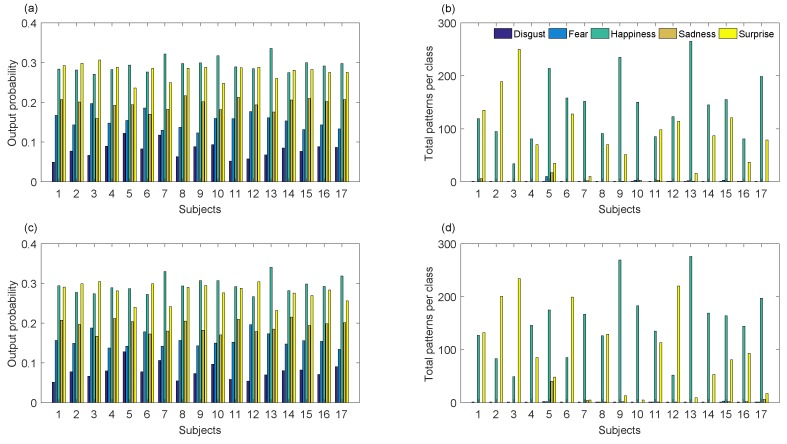
Automatic emotion recognition over unknown patterns. (**a**,**b**) are inferred emotions obtained during the Baseline phase of the experiment; (**c**,**d**) are emotion decisions achieved for the Test phase of the experiment.

**Figure 10:**
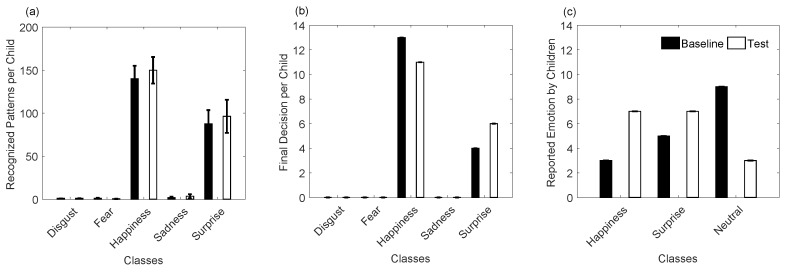
Summary of children emotions before and after they saw the robot for the first time. (**a**) and (**b**) are inferred emotions by the recognition system; (**c**) emotions reported by the children.

Similarly, this database was used as a training set of our recognition system to infer emotions on 17 children, while they firstly remained in front of N-MARIA covered by a period of time, and after it was uncovered. Figure 9a–d and Figure 10a,b show inferred emotions by our system for the seventeen children during the Baseline and Test experiments, being happiness and surprise the most frequent outputs. Emotions such as disgust, fear, and sadness were few inferred by the system, which agree with what the children reported, such as shown in Figure 10c.

Figure 10c shows that a neutral emotion was reported by more children for N-MARIA covered, while they felt surprise and happiness seeing N-MARIA by first time. In contrast, happiness and surprise were slightly more inferred after having uncovered the robot for the children, which also agreed with what the children reported. It is worth noting that emotions may vary among children for a same visual stimulus, such as a social robot. For this reason, a single indicator for evaluation is not sufficient to interpret accurately the responses of children to a robot while they are interacting. Then, a questionnaire was answered by each child, reporting their emotions during both Baseline and Test (see Section 3.1). However, self-assessments have problems with validity and corroboration, as described in Ref. [1], as participants might report differently from how they are actually thinking or feeling. Then, it is not trivial to attribute the children responses to their true behaviours. Similarly, the emotion produced by each child may be conditioned, as they know that they are being observed by the robot, parent, and the specialist conducting the experiment. Also notice that an existent database of 28 typically developing children (age: 7–11 years) was used in our study to train our proposed system to infer one of five basic emotions (disgust, fear, happiness, sadness, surprise) produced by 17 children during the interaction with N-MARIA. However, visual and thermal image processing may be affected due to the quality of the input image, as it depends of the illumination conditions and the distance between child and robot during the interaction.

## 5. Discussion

Several studies have been addressed to recognize some of the six accepted emotions by psychological theory: surprise, fear, disgust, anger, happiness and sadness [10,53,54,55]. For instance, facial motion and the tone of the speech have shown in recognition systems their relevant role to infer these aforementioned emotions, achieving accuracy (ACC) from 80% to 98% [56,57,58] and from 70% to 75% [53,59,60], respectively. Although facial expressions provide important clues about emotions, it is necessary to measure, by optical flow, the movements of specific facial muscles through markers located on the face [53,57,58]. However, this non-contact free technique may not be comfortable to study or infer emotions of children with ASD, as these children present high skin sensitivity.

Following this approach of non-contact techniques, there are some APIs (Application Program Interface) that allow facial detection, such as the “Emotion API” from Microsoft or the Oxford API [61,62]. Some works, alternatively, use other image processing techniques, such as finding the region of the thermogram within a temperature range to detect the face [18]. Another approach is to keep the face in a fixed position using a support for chin or headrest device [41,63]. Furthermore, in Ref. [64] the authors show a solution using neural network and supervised shapes classification methods applied to facial thermogram.

Emotion recognition systems based on IRTI have, in fact, shown promising results. Table 5 shows some studies that use ROI replacement and further emotion analysis, although using other techniques in comparison with our proposal. Unfortunately, the results presented are somewhat disperse, and it is not possible to make a fair comparison among them, due to the different pictures used in the studies. In Ref. [18] the authors proposed techniques for selecting facial ROIs and classify emotions using a FLIR A310 camera. To detect the face, thermogram’s temperatures between 32 ${}^{\circ }$C to 36 ${}^{\circ }$C were used to define the face position. The ROIs positions were further calculated by proportions based on the head’s width. All other temperature points were considered background. Additionally, for emotion classification, the system was calibrated using a baseline (neutral state) that compensates the induced emotion by applying fuzzy algorithm, and thus, calibrate the induced emotion image. Using the baseline, the temperature is inferred by IF-THEN rules to calibrate the thermal images for the following induced emotions: joy, disgust, anger, fear and sadness. Next, a top-down hierarchical classifier was used to analyze the emotion classification, reaching 89.9% of success rate.

The functional Infrared Thermal Imaging (fITI) was used in Refs. [21,63], which is considered to be a promising technique to infer emotions through autonomic responses. Similarly, another study was carried out using fITI to compare subjective ratings of displayed pictures for the volunteers [63], where these pictures were categorized into unpleasant, neutral and pleasant. Then, while the volunteers were watching these pictures, the authors collected the nose tip temperature (there was a chin support to keep the face correctly located in the camera image), which is one of the most likely places to change temperature when the person is under some kind of emotion [17]. As a result, they found that pictures that evoke emotions (no matter if it is a positive or a negative emotion) were more susceptible to produce thermal variation, while the difference for the neutral images was not as great as the others. Thus, their findings demonstrate that fITI can be a useful tool to infer emotions in humans.

Another interesting research [68] locates facial points in visual grayscale image using Gabor features based boosted classifiers, in which the authors used an adapted version of Viola-Jones algorithm, using GentleBoost instead of AdaBoost, to detect the face. Also, Gabor wavelet was used for feature extraction, detecting 20 ROIs that represent the facial feature points. All this detection was made automatically and contact-free using the iris and mouth detection. These two parts were detected by dividing the face in two regions, and calculating proportions to find those regions (iris and mouth). From this, all other ROIs were calculated using proportions. Their success rate was high, since the algorithm achieved a 93% of success rate using the Cohn-Kanade database, which has expressionless pictures of 200 people. Although Gabor wavelet transform is a representative method to extract local features, it takes a long time and has a large feature dimension.

Another method was proposed in Ref. [65], where a deep Boltzmann machine (DBM) was applied to recognize emotions from thermal facial images, using a previous database and with the participation of 38 adult volunteers. Their evaluation consisted of finding the emotion valence, which could be positive or negative, and their accuracy rate reached 62.9%. In their study, since the face and the background have different temperatures, they were split by applying the Otsu threshold algorithm in order to binarize images. Then, the projection curves (both vertical and horizontal) were calculated to find the largest gradient and detect the face boundary.

Additionally, a model for expression recognition using thermal images of an adult volunteer was applied in Ref. [66]. These authors used eigenfaces for feature extraction of the volunteer’s facial images through PCA to recognize five emotions (happiness, anger, disgust, sad and neutral). As a highlight, that proposal reached an accuracy close to 97%, in which work, they applied eigenvalues and eigenfaces, trained the system with a set of images, used PCA to reduce the dimensionality, and distance classifier to recognize the emotion.

In Ref. [13], authors were able to achieve 81.95% of accuracy using histogram feature extraction combined with multiclass SVM over thermal images of 22 volunteers in the Kotani Thermal Facial Expression (KTFE) database, and four classes were studied: happiness, sadness, fear and anger. They used preprocessing techniques to prepare the image to apply Viola-Jones and for further image enhancement de-noising the image and using the Contrast Limited Adaptive Histogram Equalization. For ROI detection, a ratio-based segmentation was used.

Moreover, the recognition of both baseline and affective states was carried out in Ref. [41], where, to detect the face, they used a headrest to keep it in the correct position, in addition to a reference point (located on the top of the head), which was about 10 ${}^{\circ }$C cooler than the skin temperature. To find the ROIs, the reference point was used, and a radiometric threshold was applied. In case of loss of the reference point, it was manually corrected by the researchers.

Another study [67] applied IRTI in a female adult volunteer, and Neural Networks and Backpropagation algorithms were used to recognize emotions, such as happiness, surprise and neutral state, reaching an ACC of 90%. To find the face they used Otsu segmentation, and the Feret’s diameter was found in the binary image together with the center of gravity of the binary image. Then, after segmenting the image, positions of the face based on FACS-AU were used to determine the heat variation and, thus, the emotion.

Another work [61] shows a study about several approaches of emotion recognition and facial detection, such as machine learning and geometric feature-based process, in addition to SVM and a diversity of other classifiers. They also present the use of Microsoft HoloLens (MHL) for detecting human emotions by using an application that has been built to use MHL to detect faces and recognize emotion of people facing it. The set of emotions they worked was composed of happiness, sadness, anger, surprise and neutral. Additionally, they used a webcam to detect emotions and compare with the result using MHL. The system with MHL could achieve much better results than previous works and had remarkable accuracy probably due to the sensors attached to the HoloLens, reaching an accuracy of 93.8% on MHL, using the “Emotion API” from Microsoft.

In Ref. [62], the authors used a parrot-inspired robot (KiliRo) to interact with ASD children by simulating a set of autonomous behaviors. They tested the robot for five consecutive days in a clinical facility. Children’s expressions while interacting with the robot were analyzed by the Oxford emotions API, allowing them to make an automated facial detection, emotion recognition and classification system.

Some works, such as Ref. [69], show the use of deep learning to detect the child face and infer the visual attention on a robot during CRI therapy. Authors used the robot NAO from Softbank Robotics, which has two low-resolution cameras that were used to take pictures and record videos. They also used the face detection and tracking system in-built in NAO for the clinical experiments. A total of 6 children participated of the experiment, in which they imitated some robot movements. The children had 14 encounters over a month, and the actual experiments started 7 days after the preliminary encounter, in order to avoid the novelty effect in the results. Different deep learning techniques and classifiers were used, and they could reach an average children attention rate of 59.2%.

Deep-learning-based approaches have shown to be promising for emotion recognition, determining features and classifiers without expert supervisors [10]. However, conventional approaches are still being studied for use in real-time embedded systems because of their low computational complexity and high degree of accuracy [70], although for these systems the methods for feature extraction and classification should be designed by the programmer and cannot be optimized to increase performance [71]. Moreover, it is worth mentioning that conventional approaches require relatively lower computing power and memory than deep learning-based approaches [10]. Similarly, Gabor features are very popular for facial expression classification and face recognition, due to their high discriminative power [72,73], but the computational complexity and memory requirement make them less suitable for a real time implementation.

Our system is composed of low-cost hardware and methods of low-computational cost for visual and thermal image processing, and recognizes five emotions, achieving 85.75% of accuracy. For our system, we proposed a method based on probability error to accurately locate subject-specific landmarks, taking into account the trained expert criteria. As a highlight, our proposal can find frame-to-frame the best located facial ROI using the Viola-Jones algorithm, and adjust the location of its surrounding facial ROIs. As another novelty, our proposal based on probability error showed robustness and good accuracy to locate facial ROIs on thermal images, which were collected while typically developing children interacted with a social robot. As other findings, we extended an existing database of five facial emotions from thermal images, to infer unknown emotion generated while the children interacted with the social robot, using our recognition system based on PCA and LDA, thus, achieving results that agreed with the written reports of children.

As limitation, our system is not able to track head movements, thus, adding a method for facial tracking, such as done by Ref. [74], can make robust our proposal for facial landmarks in uncontrolled scenarios, such as mobile applications for child and social robot interaction. Generally, facial emotion datasets with six basic emotions contain only adult participants, but there are very few databases collected on typically developing children (aged between 7 and 11 years) through infrared camera, containing the basic emotions. Then, it is a challenge to use high quantity of examples during the training stage of a recognition system to infer emotions of children with age range from 7 to 11 years while they interact with a robot for example. In addition, more tests with a higher number of volunteers must be performed, including ASD children.

## 6. Conclusions

A low-computational cost system for children emotion recognition in an unobtrusive way was proposed in this study, which is composed of low-cost cameras, making possible its extension for research into developing countries. As a first stage, our proposal was tested on visual and thermal images of children interacting with the mobile social robot N-MARIA, achieving promising results (85.75% of accuracy) to locate specific face landmarks, as well as to recognize (or infer) five emotions. All children had a hopeful interaction with the robot, which demonstrated our system is useful to stimulate positive emotions in children, and able to trigger a profitable interaction with them. In future works, this proposal will be integrated in N-MARIA, aiming to know online the children’s emotion and making control decisions based on emotions. Additionally, other methods will be explored for facial tracking, in order to reduce the influence of head-pose during the emotion recognition. Furthermore, unsupervised methods for automatic label assignments and classifier learning will be evaluated in our dataset to obtain a robust recognition system for processing patterns of high uncertainty, such as facial expressions and emotions.

## Figures and Tables

**Table 1:** Reference ROIs used to locate face landmarks over a frame $i_{b}$.

Reference ROIs	Located ROIs
Head	$R_{b}^{1}$, $R_{b}^{2}$, $R_{b}^{10}$, $R_{b}^{11}$
Eyes	$R_{b}^{1}$, $R_{b}^{2}$, $R_{b}^{6}$, $R_{b}^{7}$, $R_{b}^{8}$, $R_{b}^{9}$
Nose	$R_{b}^{3}$, $R_{b}^{4}$, $R_{b}^{5}$, $R_{b}^{10}$, $R_{b}^{11}$

**R**${}_{b}^{1}$, right forehead side; **R**${}_{b}^{2}$, left forehead side; **R**${}_{b}^{3}$, right periorbital side; **R**${}_{b}^{4}$, left periorbital side; **R**${}_{b}^{5}$, tip of nose; **R**${}_{b}^{6}$, right cheek; **R**${}_{b}^{7}$, left cheek; **R**${}_{b}^{8}$, right perinasal side; **R**${}_{b}^{9}$, left perinasal side; **R**${}_{b}^{10}$, right chin side; **R**${}_{b}^{11}$, left chin side.

**Table 2:** Features computed in each ROI.

Features	Equations
1. Mean value of the whole ROI	$f_{bc}^{k}=\bar{R_{b}^{k}}=\frac{1}{m\cdot n}\sum_{i=1}^{m}\sum_{j=1}^{n}R_{ij}$, c=1
2. Variance form the whole ROI, organized in a vector	$f_{bc}^{k}=\sigma_{b}^{2k}=\frac{1}{(m\cdot n)-1}\sum_{i=1}^{m}\sum_{j=1}^{n}{\left(R_{ij}-\bar{R_{b}^{k}}\right)}^{2}$, c=2
3. Median of the whole ROI, organized as a vector	$f_{bc}^{k}=median\left(\bar{R_{b}^{k}}\right)$, c=3
4. Mean of variance values in rows	$f_{bc}^{k}=\frac{1}{m}\sum_{i=1}^{m}\frac{1}{n-1}\sum_{j=1}^{n}{\left(R_{ij}-\bar{R_{bi}^{k}}\right)}^{2}$, c=4
5. Mean of median values in rows	$f_{bc}^{k}=\frac{1}{m}\sum_{i=1}^{m}median\left(R_{bi}^{k}\right)$, c=5
6. Mean of variance values in columns	$f_{bc}^{k}=\frac{1}{n}\sum_{j=1}^{n}\frac{1}{m-1}\sum_{i=1}^{m}{\left(R_{ij}-\bar{R_{bj}^{k}}\right)}^{2}$, c=6
7. Mean of median values in columns	$f_{bc}^{k}=\frac{1}{n}\sum_{j=1}^{n}median\left(R_{bj}^{k}\right)$, c=7
8. Difference from each item in consecutive frames	$f_{b(c+7)}^{k}=f_{bc}^{k}-f_{(b-1)c}^{k}\;,\;c=1,2,\cdots ,7$

**Table 3:** Performance of the system for five emotions recognition using FNCA plus LDA based on full covariance matrices.

	Disgust	Fear	Happiness	Sadness	Surprise
TPR (%)	90.02 ± 1.91	87.25 ± 2.37	87.55 ± 2.12	74.69 ± 2.77	86.93 ± 2.12
FPR (%)	3.07 ± 0.49	4.17 ± 0.57	4.61 ± 0.69	3.42 ± 0.78	2.93 ± 0.50
ACC (%)	85.29 ± 1.16				
Kappa (%)	81.26 ± 1.46				

ACC, accuracy; TPR, true positive rate; FPR, false positive rate.

**Table 4:** Performance of the proposed system for five emotions recognition using PCA with 60 components plus LDA based on full covariance matrices.

	Disgust	Fear	Happiness	Sadness	Surprise
TPR (%)	89.93 ± 1.88	88.22 ± 2.38	86.36 ± 2.09	76.15 ± 2.80	87.14 ± 2.15
FPR (%)	2.80 ± 0.47	3.48 ± 0.47	5.40 ± 0.83	3.42 ± 0.81	2.42 ± 0.44
ACC (%)	85.75 ± 1.16				
Kappa (%)	81.84 ± 1.46				

ACC, accuracy; TPR, true positive rate; FPR, false positive rate.

**Table 5:** Comparison among some strategies used to infer emotions using Infrared Thermal Imaging (IRTI).

Studies	Volunteers	Age	ACC (%)	Summary
Cruz-Albarran et al. [18]	25	19 to 33	89.90	Fuzzy algorithm, IF-THEN rules and top-down hierarquical classifier. The analyzed emotions were joy, disgust, anger, fear and sadness.
Basu et al. [13]	26	11 to 32	87.5	Histogram feature extraction and multiclass support vector machine (SVM). The Kotani Thermal Facial Expression database was used to detect emotions of anger, fear, happiness, and sadness.
Nhan and Chau [41]	12	21 to 27	80.0	Comparison between baseline and affective states. High and low arousal and valence are compared with the baseline.
Wang et al. [65]	38	17 to 31	62.90	Deep Boltzmann Machine to find positive or negative valence.
Bijalwan et al. [66]	1	N/A	97.0	Model for expression recognition in thermal images. Application of PCA for recognizing happiness, anger, disgust, sadness and neutral emotions.
Yoshitomi et al. [67]	1	N/A	90.0	Neural Networks and Backpropagation algorithms that recognize emotions of happiness, surprise and neutral state.
Kosonogov et al. [63]	24	20 to 24	N/A	Studied tip of nose thermal variation in volunteers with images from International Affective Picture System (IAPS) which found that positive or negative images showed more temperature change compared with neutral images
Vukadinovic and Pantic [68]	200	18 to 50	N/A	Algorithm to find facial ROIs. A Viola-Jones adapted algorithm (that applies GentleBoost instead of AdaBoost) was used. For facial feature extraction Gabor wavelet filter was used. No emotion classes were studied, only the facial points for ROI detection.
Bharatharaj et al. [62]	9	6 to 16	N/A	AMRM (indirect teaching method) was studied using a parrot-inspired robot and the Oxford emotion API to recognize and classify emotions in ASD children. Most of them appeared to be happy with the robot.
Mehta et al. [61]	3	N/A	93.8%	Microsoft HoloLens (MHL) system was used to detect emotions achieving a high accuracy to detect happiness, sadness, anger, surprise and neutral emotions.
Our proposal	28	7 to 11	85.75%	PCA and LDA were used on our database, published in [17], to recognize happiness, sadness, fear, surprise and disgust.

ACC, accuracy; N/A means that the age or ACC were not reported.
